# A Method for Automated Classification of Parkinson’s Disease Diagnosis Using an Ensemble Average Propagator Template Brain Map Estimated from Diffusion MRI

**DOI:** 10.1371/journal.pone.0155764

**Published:** 2016-06-09

**Authors:** Monami Banerjee, Michael S. Okun, David E. Vaillancourt, Baba C. Vemuri

**Affiliations:** 1 Department of CISE, University of Florida, Gainesville, Florida, United States of America; 2 Department of Neurology, University of Florida, Gainesville, Florida, United States of America; 3 Center for Movement Disorders and Neurorestoration, University of Florida, Gainesville, Florida, United States of America; 4 Department of Applied Physiology and Kinesiology, University of Florida, Gainesville, Florida, United States of America; University of Ulm, GERMANY

## Abstract

Parkinson’s disease (PD) is a common and debilitating neurodegenerative disorder that affects patients in all countries and of all nationalities. Magnetic resonance imaging (MRI) is currently one of the most widely used diagnostic imaging techniques utilized for detection of neurologic diseases. Changes in structural biomarkers will likely play an important future role in assessing progression of many neurological diseases inclusive of PD. In this paper, we derived structural biomarkers from diffusion MRI (dMRI), a structural modality that allows for non-invasive inference of neuronal fiber connectivity patterns. The structural biomarker we use is the ensemble average propagator (EAP), a probability density function fully characterizing the diffusion locally at a voxel level. To assess changes with respect to a normal anatomy, we construct an unbiased template brain map from the EAP fields of a control population. Use of an EAP captures both orientation and shape information of the diffusion process at each voxel in the dMRI data, and this feature can be a powerful representation to achieve enhanced PD brain mapping. This template brain map construction method is applicable to small animal models as well as to human brains. The differences between the control template brain map and novel patient data can then be assessed via a nonrigid warping algorithm that transforms the novel data into correspondence with the template brain map, thereby capturing the amount of elastic deformation needed to achieve this correspondence. We present the use of a manifold-valued feature called the Cauchy deformation tensor (CDT), which facilitates morphometric analysis and automated classification of a PD versus a control population. Finally, we present preliminary results of automated discrimination between a group of 22 controls and 46 PD patients using CDT. This method may be possibly applied to larger population sizes and other parkinsonian syndromes in the near future.

## Introduction

Parkinson’s disease (PD) is a neurodegenerative disorder that impairs movement, mood, autonomic functions, cognition and quality of life. PD can cause considerable difficulty with daily tasks such as grooming, walking, and other activities of daily living. PD affects millions of people across the globe, and estimates project that the number of people with PD will continue to grow [1,2]. To improve therapeutic development, the field will require viable markers of disease that can be measured in both humans with PD. Additionally, markers of disease are needed in animal models of PD neurodegeneration. Mathematical tools and computational algorithms using advanced non-invasive imaging technologies across human and animal models of PD could potentially facilitate improved brain mapping in this population.

A promising technique that may be able to achieve clinically relevant separation in PD and controls is the use of diffusion MRI [3]. Diffusion MRI is a noninvasive way to probe the axonal fiber connectivity in the body by making the MR signal sensitive to water diffusion through tissue [4]. It is however, well known that DTI cannot resolve complex fiber geometries e.g., crossing and splaying fibers that occur commonly in the brain. Most importantly, the use of the single tensor (DTI) model has led to a wide variability in the reported results in the literature [5], leading to failures in detecting key differences in PD patients. In contrast, high angular resolution diffusion imaging (HARDI) provides the required resolution and when used in conjunction with sophisticated mathematical modeling techniques, captures the complex local fiber geometries and changes due to the underlying pathology. To capture the full diffusional information contained in the HARDI data, we proposed to use the EAP [6], the “holy grail” of diffusion MRI (dMRI) processing. The EAP represents a probability density function which captures the local multi-fiber geometry. Several methods exist in the literature to estimate the EAP fields from the HARDI data [7–10]. We will use the method developed and previously published by our group [8, 11] and apply it to both humans with PD. The method could potentially in the future be used to study rodent and non-human primate models of PD associated neurodegeneration but for this study we focused on human subjects. To establish a basis for comparison between the controls and PD patients, we present a novel method to construct multiple intrinsic brain map templates of EAP fields from a heterogeneous population of HARDI brain scans of control patients. An intrinsic template brain map is a population specific template that is an intrinsic average over the population of the given EAP fields.

## Methods

Many research groups have been focused on DTI to achieve clinically relevant separation between PD and controls through the use of a simple biomarker derived from diffusion MRI data [3, 12, 13]. In two recent papers, [12, 13] using DTI based measures, PD patients were found to exhibit significantly reduced fractional anisotropy (FA) as compared to controls, suggesting DTI as a potential early trait PD biomarker. Several other studies have reported reduced nigral FA values in pharmacologically treated PD patients as compared to controls, but none achieved complete group separation [1, 14, 15]. This relatively lower sensitivity and specificity may be caused by disease effects, medication status, and/or methodological differences such as scanner field strength, ROI placement/size, number of gradient directions or the inadequateness of the diffusion tensor representation to capture complex local tissue geometries such as crossing and kissing axon fiber geometries. To capture the information about these complex fiber geometries and to develop more sensitive biomarkers would require HARDI. Existing biomarkers for PD detection ignore orientation information, and new biomarkers are needed to capture both shape and orientation information contained in the dMRI signal via the use of the EAPs, which may increase sensitivity and specificity of PD detection.

More specifically, EAPs will capture the full anisotropy information including the directional information. EAPs are a rich source of information, fully characterizing the local diffusion process at a voxel in the acquired data. Regardless of the type of diffusion sensitized MR signal decay model used, EAPs by definition can be computed with ease and capture the full (local) anisotropy information and will be limited only by the signal decay model utilized. For example, the shape of EAP will change if there is a decrease/increase in anisotropy and it is directionally sensitive. This is a very powerful property of EAPs and it occurs independent of the neurological disorder under consideration. Thus, regardless of the neurological disorder, EAPs can be used to capture changes in tissue architecture caused by the disorder and hence potentially serve as a useful biomarker.

### Proposed Method of Brain Mapping for use in Parkinson’s Disease

Here we present a novel unbiased population specific brain map template construction method from the given EAP-fields. The method however will be applicable to any other type of images in general. In diffusion weighted MRI, the water diffusion is fully characterized by the diffusion Probability Density Function (PDF) called the ensemble average propagator (EAP) [6]. Under the narrow pulse assumption, the EAP denoted by *P*(**r**) and the diffusion signal attenuation *E*(**q**) are related through the Fourier transform [6], *P*(**r**) = ∫ *E*(**q**) exp(−2*πi***q** ∙ **r**) d**q** where, *E*(**q**) = *S*(**q**)/*S*_0_, *S*_0_ is the diffusion signal with zero diffusion gradient, **q** is the vector along which the diffusion gradient is applied and **r** is the radial vector in the dual space defined through the Fourier relationship above. *P*(**r**) at each voxel, captures all the information needed to perform tractography since it is well known that the peaks of this distribution correspond to the local fiber orientations.

There are several methods in the literature to compute the *P*(**r**) and we adopted a method developed by our group [8, 11]. This approach when applied to the input HARDI data yields an EAP field/image. Given a control population of HARDI data, by applying the algorithm we get a population of EAP-fields. We use this set of EAP fields to construct an unbiased population specific template brain map. A template brain map of a population of images/shapes is commonly defined as an average over the population, which is taken to be a representative of the population. The problem with simply taking an average as the template brain map is that the average will be rather blurred and is not effective for use in tasks such as template-based segmentation or registration. This happens since the average in general may not necessarily belong to the same abstract space (e.g., space of brain images) defined by the original data set. To solve this problem the template brain map was constrained to be deformed diffeomorphically from a super template [16], which was pre-selected. In [17–19], the geometry of the subject’s image space is learned from the dataset, and then used to compute the template brain map.

A DTI based template brain map will obviously provide more information than a conventional scalar image based template brain map since DTI contains both scalar and directional information [20]. The brain map template construction requires the DTI data to be group-wise registered, and prior work has used different approaches for this unique problem [21, 22]. The ICBM-DTI-81 template registration techniques reported in the literature were pairwise registration methods [23–27]. Some of the existing DTI based brain map templates are built by using a brain map technique [20] and perhaps provide the most widely used reference coordinate system for group analyses of diffusion tensor MR images. However, it was pointed out that there were several substantial problems with this template brain map including correctness of spatial orientations and white matter tract labels [28].

It is however well known that the DTI model cannot resolve complex tissue structure such as fiber crossings. To handle this problem, several higher order models [11, 29–34] based on HARDI dataset have been recently reported in the literature. Several researchers have addressed the HARDI pairwise registration problem using high rank tensors or orientation distribution functions (ODFs) for representing this data, [35–39], and show that their methods outperform DTI based registration especially in aligning fiber crossing regions [35]. More recently, a few researchers have reported group-wise registration of HARDI datasets using, a 4-th order tensor field representation [36], which extends the unbiased template brain map construction technique in [40]. In [41], Du et al. developed a Bayesian framework for the template brain map construction from ODF fields estimated from the HARDI scans. The Bayesian framework allowed them to explicitly incorporate prior anatomical shape information. Finally, there are a few methods that resort to building the template brain map directly from the population of HARDI data without committing to any representation of the diffusion MR signal [42, 43]. In these methods, the nonlinear registration is achieved using the FA maps in the former and the raw **q**-space data in the latter. Note that FA is a scalar feature that ignores the orientation information contained in the diffusion signal and will possibly lead to erroneous registrations.

All of these methods lead to “unsharp” template brain maps, which is basically due to the fact that “averaging” blurs the details. In our work [44], we define the space of images of interest to us (diffusion MRI scans of the brain) to be spanned by a set of Gaussian mixture fields (GMFs)—each GMF represents an EAP field -, ${\left\{I_{n}\right\}}_{n=1}^{N}$ and denoted by *S* = ⋃_*n*_ *O*(*I*_*n*_), where *O*(*I*_*n*_) = {*J*:*J* = *I*_*n*_ ∘ ***T***,***T*** ∈ *Diff*} is the orbit spanned by the image *I*_*n*_ and the diffeomorphic deformations $T_{n}:Ω\to {Ω}_{I_{n}}$, where Ω denotes the domain of the image. Thus, finding the template brain map, *I*, can be viewed as solving the following optimization problem $m^{*},T_{1}^{*},\cdots =arg\;\min_{m,T_{1},\cdots }\sum_{n}E(I_{n}\circ T_{n},I_{m}\circ T_{m})+\emptyset (T_{n})$, and the final template brain map can be defined as $\hat{I}=\Phi_{m^{*}}^{-1}[I_{m^{*}}\circ T_{m^{*}}]$, where $\Phi_{m^{*}}$ is the Jacobian of the deformation $T_{m^{*}}$, and $\Phi_{m^{*}}^{-1}[∙]$ denotes the re-orientation operation discussed previously [44]. Preservation of Principal Direction (PPD) re-orientation is extended to Gaussian Mixtures (GMs) in [37], and it is the only re-orientation strategy that can capture the change of angle between fiber crossings during a non-rigid registration transformation. In this work, we adopt this re-orientation strategy. Solving this problem directly would make the computational complexity similar to *O*(*N*^2^) pairwise registrations. What we do instead is to achieve an approximate solution using a two-stage approach.

The first involves finding an intermediate template brain map in the space of all images, which can be cast as an optimization problem (similar to the approach in [40] but generalized to GMFs) $I,T_{1},\cdots =arg\;\min_{I,T_{1},\cdots }\sum_{n}E(I_{n}\circ T_{n},I)+\emptyset (T_{n})$ where, *E*(,) in this work is defined as a sum of squared voxel-wise distances (see [44])

$$E(I_{n}\circ T_{n},I)=\int_{{Ω}_{I}}{dist}_{\Phi_{n}}^{2}(I_{n}\circ T_{n}(x),I(x))\;dx,$$ (1)

and ∅ penalizes lack of smoothness in the deformation. The deformation is modeled as a diffeomorphism and parameterized by a velocity field $\frac{\partial T_{n}}{\partial t}=v_{n}(T_{n}(x,t),t)$. The deformation is thus computed as $T_{n}(x)=x+d_{n}(x)=x+\int_{0}^{1}v_{n}(x(t),t)\;dt$, where **d**_*n*_ represents the displacement field. The smoothness constraint we used is given by,

$$\emptyset \left(T_{n}\right)=\lambda *\log\left(\text{det}\left(\Phi_{n}\right)\right)*\left(1-\text{det}\left(\Phi_{n}\right)\right)+\int_{{Ω}_{I}}\int_{0}^{1}{\left\|Lv_{n}\left(x,t\right)\right\|}^{2}\;dx\;\text{d}t,$$ (2)

where, *L* is a linear operator, while the first term in Eq 2 corresponds to additional smoothness imposed as in [45]. This stage requires O(N) registrations.

The second step involves projecting the intermediate template brain map to the space *S* by solving another distance minimization given by,

$$\hat{I}=\Phi_{m^{*}}^{-1}[I_{m^{*}}\circ T^{*}]$$

$$m^{*},T^{*}=\text{arg}\;\min_{n,T}E\left(I_{n}\circ T,I\right).$$ (3)

The projection $\hat{I}$ then is our final template brain map and this step also needs O(N) registrations. Overall, this two stage method needs O(N) registrations as opposed to O(N^2^) using the direct method of atlas construction, making it computationally more efficient.

### Subject Characterization

Sixty eight individuals participated in this study: 46 PD and 22 controls. All PD patients were diagnosed by a fellowship-trained movement disorders specialist using established criteria, and all were Hoehn and Yahr stage 2–3 off medication. None of the control participants reported a history of neuropsychiatric or neurological problems. Research evaluations were conducted between 9:00 am and 3:00 pm, and the patients were tested following overnight withdrawal from anti-Parkinsonian medications. All participants were administered the Movement Disorder Society Unified Parkinson’s Disease Rating Scale part III (MDS-UPDRS-III) to evaluate motor symptom severity, Beck’s Depression Index (BDI) to assess depression, and the Montreal Cognitive Assessment (MoCA) to assess global cognitive functioning. The study was approved by the Institutional Review Board at the University of Florida. Informed written consent was obtained from each subject prior to testing.

The mean age of the control group was 64.6 years (SD = 10.2), and PD group was 64.2 years (SD = 8.6). Mean MDS-UPDRS-III for the control group was 3.2 (SD = 2.8) and the PD group was 25.84 (SD = 10.41). MoCA and BDI for the control group were 26.95 (SD = 2.0) and 5.0 (SD = 5.1), and for the PD group were 25.8 (SD = 2.5) and 7.6 (SD = 5.9), respectively. Independent samples t-test for age, MoCA, and BDI did not approach significance (p’s > 0.25). As expected, MDS-UPDRS-III was greater in the PD group compared with the controls (t = 9.9; p < 0.001). The Mann-Whitney Test was used to compare sex between groups and this did not approach significance (p > 0.9). There were 15 male and 7 female PD, and 32 male and 14 female controls.

### Data Acquisition

We now present results of using our algorithm described above to estimate the template brain map from a population of diffusion MR brain scans of controls. All subjects signed informed consent that was approved by the internal review board. HARDI scans from a population of 22 controls and 46 PD were acquired for the experiments reported in this paper. Whole brain diffusion imaging data were acquired using a single-shot spin echo EPI sequence, with repetition time = 7748 ms, echo time = 86 ms, flip angle = 90°, field of view = 224 × 224 mm, voxel size = 2 mm isotropic with no gap between slices (n = 60), diffusion gradient (monopolar) directions = 64, diffusion gradient timing DELTA/delta = 42.4/10 ms, b-values: 0, 1000 s/mm2, fat suppression using SPIR, in-plane, SENSE factor = 2.

**Fig 1:**
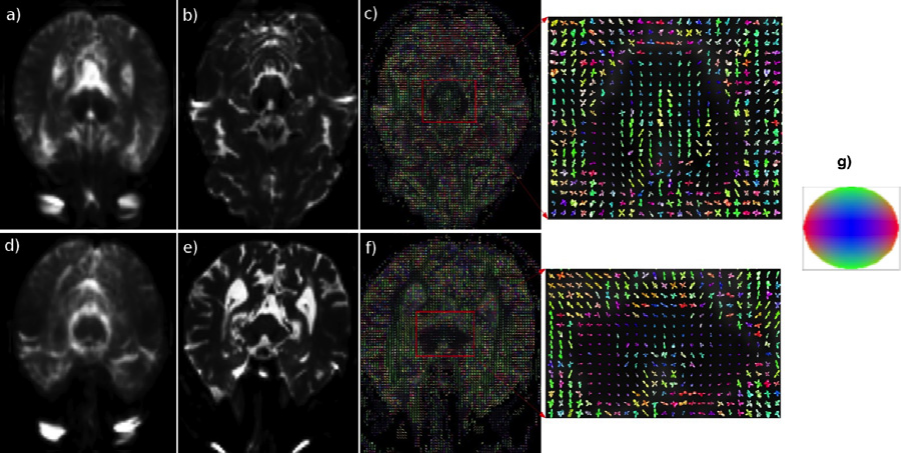
Template Brain map (EAP field). (a) and (b) are the corresponding S_0_ (zero magnetic gradient) slices of the constructed template brain map and a control, respectively. (c) The template EAP field of the same slice as in (b), with the ROI containing the Substantia Nigra (SN). (d) and (e) The corresponding S0 slices of the template and a PD patient, respectively, and (f) shows the template EAP field computed for the slice in (e), with the ROI containing the SN. (g) is the color ball used to denote the directions in the EAP fields.

The method [11] was used to estimate the EAPs—represented by Gaussian mixture models—from the MR signals at each voxel. We first apply a similarity registration to quotient out the translation, rotation and scaling factors. Then, we applied our group-wise registration algorithm presented above, and described in detail previously [44], to the dataset of 22 controls to obtain an intermediate template brain map *I*. Following that, we project *I* to the space spanned by the given data samples using Eq 3 to get the “sharp” (non-blurry) template brain map $\hat{I}$. The results of our template brain map construction method are depicted in Fig 1. For ease of visualization, we depict them using the *S*_0_ (zero-gradient) images and corresponding slices from the EAP fields. The EAPs are colored using the directions associated with the peaks of the EAPs. Here, the *XYZ* directions are mapped to red, green and blue colors respectively. X is left to right, Y is bottom to top and Z is points into the plane.

## Results

In this section, we present a classification technique that automatically discriminates the diffusion MR scans obtained from controls and PD patients. For the classification, we used 22 controls and 46 PD diffusion MR brain images, each with a resolution 112 × 112 × 60, with 64 gradient directions. The image acquisition protocol is the same as described earlier and in [46]. In order to achieve the classification, one requires a biomarker that is rich enough to capture the differences between the groups. In our case, we chose the EAP, a probability density function, at each voxel as the biomarker. We then perform EAP-based morphometry to capture the changes between the constructed template brain map and the patient EAP fields. This change can be captured by non-rigidly warping the patient EAP field to the template brain map (EAP field). The amount of elastic transformation between the two EAP fields is then characterized by the Cauchy deformation tensor field. The Cauchy deformation tensor (CDT) at each voxel is defined as the $\sqrt{JJ^{T}}$, where *J* is the Jacobian of the nonrigid/elastic transformation. The CDT thus captures the local change between the template brain map and the patient data, which is an excellent biomarker for group-wise discrimination. Since the CDTs are symmetric positive definite matrices, which live in a Riemannian manifold with negative sectional curvature, standard classification algorithms developed for Euclidean spaces are not applicable directly to the CDT fields. Further, since the dimension of each CDT, which is a symmetric (3,3) matrix, is 6 and the chosen ROI has 362 voxels, we have a 6 × 362 = 2172 dimensional classification problem and only a population of size 68 (including controls and PD patients). This is then an ill-posed classification problem. We chose to reduce the dimension down by using the nonlinear generalization of the well-known PCA algorithm called the Principal Geodesic Analysis (PGA) applied to the CDT fields using the established technique [47]. We retained 67 principal directions after applying PGA and thus the dimensionality of the problem is now reduced to 67.

Now each data point has 67 components, from which we want to select *r*-components, which can achieve a classification of PD from controls. We used the popular *v*-SVM (support vector machine) [48], with a radial basis function kernel. For an optimal performance, we performed a grid search on the two required parameters namely, the kernel function parameter, *g*, and the *ν* value for the SVM. The *g* value is varied from 0.001, to 1, with a step size of 0.001, and *ν* is varied from 0.001, to 0.90, in steps of 0.001. For classification, we used a standard leave-one-out approach. The *r* value was varied from 5 to 65 in increments of 5. The various parameter values resulting from the aforementioned selection process was, *r* = 30, *g* = 0.358, *ν* = 0.176. This lead to a classification accuracy of 98.53% with a sensitivity of 0.98 and specificity of 1.00. We also computed the fractional anisotropy (FA) from these 68 diffusion data sets, using the DTIFIT function in the FSL package [49], and performed classification of PD patients from controls, based on the same ROI. The comparison of the two classifications, using CDT and FA as biomarkers, is shown in Table 1.

**Table 1:** 

CDT			FA		
Classification accuracy	Sensitivity	Specificity	Classification accuracy	Sensitivity	Specificity
**98.53** %	**0.98**	**1.00**	76.47%	0.78	0.73

**Fig 2:**
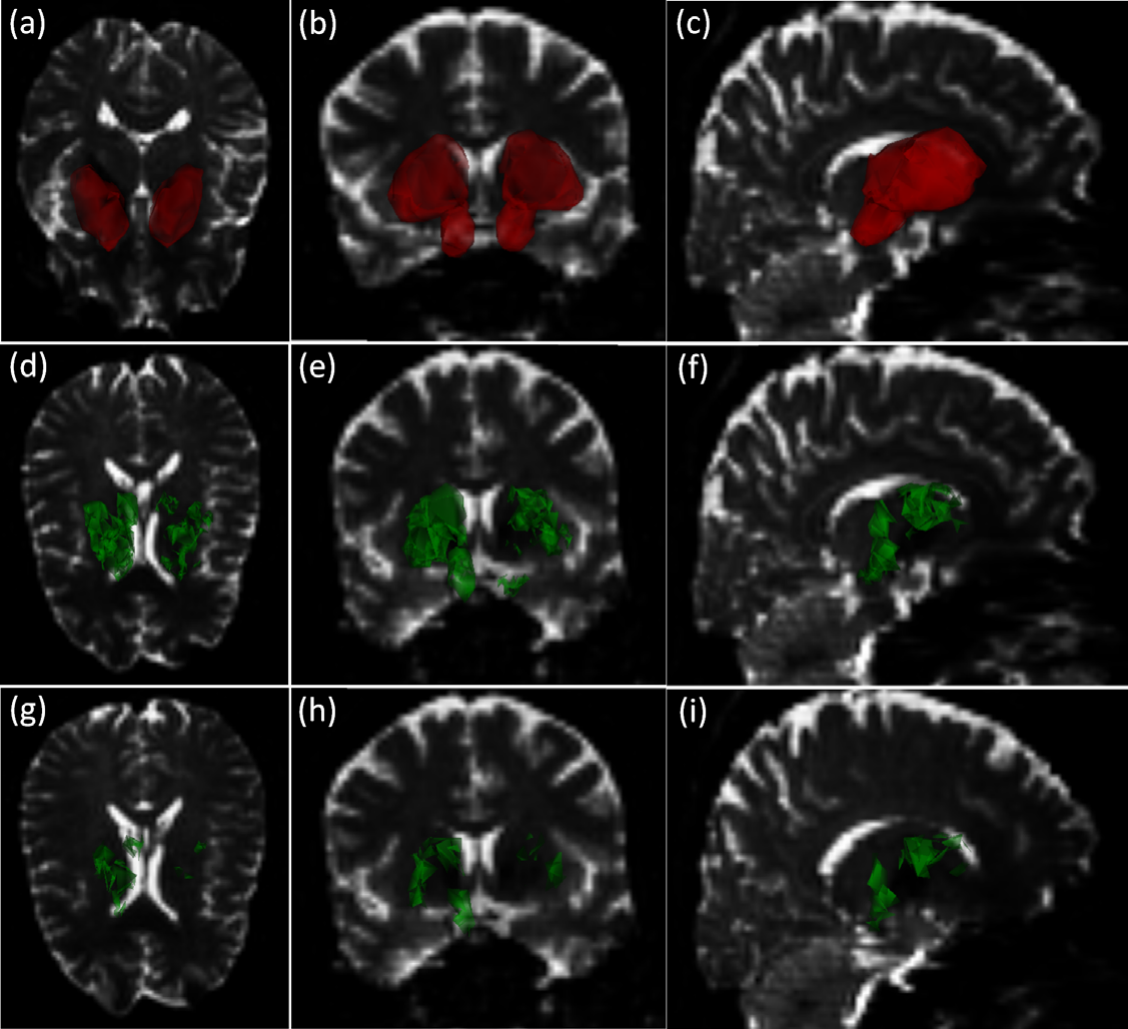
(a)—(c) are the axial, coronal, and sagittal views of the original ROI. (d)—(f), and (g)—(i) show the most discriminative voxels, with p-values < 0.05, and < 0.01, respectively.

Based on the CDT fields, we also performed a t-test on the manifold of CDTs [50] to locate the most discriminative voxels in the ROI. In Fig 2, we show all the voxels, which reject the null hypothesis, that the CDT in that voxel belongs to the same distribution for both Control and PD, with probability > 95%, and > 99%.

## Discussion and Conclusions

CDT utilized as a biomarker is a much better discriminator than FA (see Fig 1). This finding should be intuitive, since in each voxel, CDT contains both the shape and direction of the diffusion, whereas FA only captures the shape information. This result is promising and provides the data necessary to proceed and to test our method on larger population sizes and in animal models in the near future.

One key area not addressed in this study was the comparison of PD to other forms of Parkinsonism. Other forms of Parkinsonism may include multiple system atrophy and progressive supranuclear palsy. In prior work using a bi-tensor diffusion model that quantified free-water and free-water corrected fractional anistropy, specific regions of the basal ganglia, midbrain, and cerebellum were used to distinguish multiple system atrophy, progressive supranuclear palsy, and PD [51]. It remains unclear if the CDT based method developed here could provide automated discrimination of these other diseases. Given that multiple system atrophy and progressive supranuclear palsy affect the cerebellum to a greater extent than PD, it is likely the case that this structure would be needed in the original regions for the CDT based algorithm to be successful.

In this paper, we presented methods for construction of a template brain map EAP field for use in automated discrimination of PD patients from control subjects. We also presented the results of automated discrimination of PD patients vs. control subjects, using the CDT computed from the EAP fields as a biomarker and a feature selection method in conjunction with the *ν*-SVM classifier. Discrimination was achieved with 98.53% accuracy on a population size of 68. The population consisted of 46 PD patients and 22 controls. We also included a comparison with classification using FA, which showed the effectiveness of the CDT as a biomarker. Using the CDT fields, we identified the most discriminating voxels in the ROI. Our future efforts will be focused on applying these methods in much larger populations of PD patients and in testing the outcomes as well as validating them against other existing multi-site cohorts. Additionally, we plan to expand this technique for use in other parkinsonian and other neurological disorders and into animal models.
